# Genomic, biochemical and expressional properties reveal strong conservation of the *CLCA2* gene in birds and mammals

**DOI:** 10.7717/peerj.14202

**Published:** 2022-11-08

**Authors:** Florian Bartenschlager, Nikolai Klymiuk, Achim D. Gruber, Lars Mundhenk

**Affiliations:** 1 Department of Veterinary Pathology, Faculty of Veterinary Medicine, Freie Universität Berlin, Berlin, Germany; 2 Large Animal Models in Cardiovascular Research, Internal Medical Department I, Technische Universität München, Munich, Germany; 3 Center for Innovative Medical Models, Ludwig-Maximilians-Universität München, Munich, Germany

**Keywords:** Evolution, CLCA, Avian, Mammal, Chicken, Ostrich, Turkey, Quail, Keratinocyte, Skin

## Abstract

Recent studies have revealed the dynamic and complex evolution of *CLCA1* gene homologues in and between mammals and birds with a particularly high diversity in mammals. In contrast, *CLCA2* has only been found as a single copy gene in mammals, to date. Furthermore, *CLCA2* has only been investigated in few mammalian species but not in birds. Here, we established core genomic, protein biochemical and expressional properties of *CLCA2* in several bird species and compared them with mammalian *CLCA2*. Chicken, turkey, quail and ostrich *CLCA2* were compared to their mammalian orthologues using *in silico*, biochemical and expressional analyses. *CLCA2* was found highly conserved not only at the level of genomic and exon architecture but also in terms of the canonical CLCA2 protein domain organization. The putatively prototypical galline *CLCA2 (gCLCA2)* was cloned and immunoblotting as well as immunofluorescence analyses of heterologously expressed *gCLCA2* revealed protein cleavage, glycosylation patterns and anchoring in the plasma membrane similar to those of most mammalian CLCA2 orthologues. Immunohistochemistry found highly conserved CLCA2 expression in epidermal keratinocytes in all birds and mammals investigated. Our results suggest a highly conserved and likely evolutionarily indispensable role of CLCA2 in keratinocyte function. Its high degree of conservation on the genomic, biochemical and expressional levels stands in contrast to the dynamic structural complexities and proposed functional diversifications between mammalian and avian *CLCA1* homologues, insinuating a significant degree of negative selection of *CLCA2* orthologues among birds and mammals. Finally, and again in contrast to *CLCA1*, the high conservation of *CLCA2* makes it a strong candidate for studying basic properties of the functionally still widely unresolved *CLCA* gene family.

## Introduction

*Chloride channel regulators, calcium activated* (*CLCA*) constitute a family of genes that has been correlated to various disease conditions, including chronic inflammatory airway diseases (Hoshino et al., 2002; Nakanishi et al., 2001; Patel, Brett & Holtzman, 2009; Range, Mundhenk & Gruber, 2007), cystic fibrosis (Hauber et al., 2003; Ritzka et al., 2004; Young et al., 2007) and cancer (Chen et al., 2019; Hou et al., 2017; Walia et al., 2009; Walia et al., 2012; Yu, Walia & Elble, 2013) and shows striking evolutionary dynamics (Bartenschlager et al., 2022; Mundhenk et al., 2018). In general, CLCAs comprise a prototypical protein domain architecture of a CLCA N-terminal (N-CLCA), a von Willebrand factor type A (vWA), a beta sheet rich (bsr) as well as a carboxy (C)-terminal fibronectin type III domain (fn3) that is separated from N-CLCA, vWA and bsr by proteolytical cleavage (Patel, Brett & Holtzman, 2009). Some CLCAs contain a transmembrane (TM) domain that anchors the C-terminal cleavage product in the plasma membrane (Bartenschlager et al., 2022; Braun et al., 2010; Elble et al., 2006; Patel, Brett & Holtzman, 2009; Plog et al., 2012a). CLCAs lacking the TM appear completely secreted (Gibson et al., 2005; Mundhenk et al., 2006; Patel, Brett & Holtzman, 2009; Plog et al., 2009).

On the genomic level, all *CLCA* genes in a given species are located within in a single locus, which is consistently flanked by the *Outer dense fibre of sperm tails 2-like* (*ODF2L*) and *SH3-domain GRB2-like endophilin B1* (*SH3GLB1*) genes (Bartenschlager et al., 2022; Mundhenk et al., 2018; Plog et al., 2009). Although *CLCA* genes have been identified in numerous avian, reptile, amphibian or fish species in the recent past, the main research has been focused on *CLCA* homologues in mammalian species so far (Cunningham et al., 2021). From the phylogenetic perspective, mammalian *CLCA* genes have been assigned to four clearly distinct clusters (Mundhenk et al., 2018; Plog et al., 2009). While clusters 3 and 4 exhibit a complex arrangement of multiple genes that seemingly arose from several independent duplication and inactivation events during the emergence of mammalian species (Bartenschlager et al., 2022; Mundhenk et al., 2018), both *CLCA* clusters 1 and 2 comprise only one intact single gene in each mammalian species. Recently, we described a *CLCA* locus in the genome of chicken (*Gallus gallus*) which is similarly flanked by the galline *ODF2L* and the *SH3GLB1* genes (Bartenschlager et al., 2022; Mundhenk et al., 2018). In contrast to the four mammalian *CLCA* (ma*CLCA)* clusters, however, chickens possess only two *CLCA* homologous genes, the galline CLCA1 (*gCLCA1*) and *gCLCA2*. From an evolutionary perspective, *gCLCA1* groups with mammalian clusters *CLCA1/3/4* and might therefore be the avian representative of the monophyletic ancestor of this group (Bartenschlager et al., 2022). In contrast to the genetically and functionally divergent *CLCA1/3/4* cluster, g*CLCA2* seems to be much more conserved with *gCLCA2* having a close genetic relationship to ma*CLCA2* (Bartenschlager et al., 2022; Mundhenk et al., 2018).

Concordantly with the high degree of conservation, maCLCA2 is consistently expressed in keratinocytes of stratified epithelia such as those of skin or esophagus and in certain glandular epithelia in all mammals investigated so far (Braun et al., 2010; Connon et al., 2006; Connon et al., 2004; Erickson, Gruber & Mundhenk, 2020; Hamalainen et al., 2021; Plog et al., 2012b; Seltmann et al., 2018; Walia et al., 2012). It has been proposed that the CLCA2 protein is involved in epithelial differentiation, growth arrest and maturation of keratinocytes (Connon et al., 2004; Koegel & Alzheimer, 2001; Ramena et al., 2016). Some species-specific differences regarding the expression, however, have been revealed in the mammalian respiratory tract, where human, porcine, and murine, but no feline CLCA2 has been detected in airway submucosal glands (Dietert et al., 2015; Erickson et al., 2018). Further, additional protein expression has been found in a specific subset of respiratory epithelial cells of the bronchial bifurcation in mice (Dietert et al., 2015).

Aiming at clarifying the relevance of non-mammalian *CLCA2*, we characterized the genomic organization and tissue und cellular expression patterns of *CLCA2* in four bird species, including chicken (*Gallus gallus*), turkey (*Meleagris gallopavo*), quail (*Coturnix species (sp.)*), and ostrich (*Struthio camelus*). The genomic organization as well as the protein domain architecture are described for the galline, quail and ostrich orthologues. We further describe the biochemical properties of CLCA2 in chicken as a presumed avian prototype. By comparison with its porcine, feline and murine CLCA2 homologues, conserved architectural elements, specific traits of biochemical processing and tissue expression patterns were identified, which will serve as the basis for functional investigations and structure-function-correlations in the future.

## Material and Methods

### *In silico* sequence analysis of gCLCA2 and generation of antibodies

Detailed gene positions, sizes, gene and amino acid (aa) sequences from chicken, quail, ostrich, pig, cat, and mouse *CLCA* loci were extracted from the NCBI (https://www.ncbi.nlm.nih.gov/) and Ensembl (http://www.ensembl.org/index.html) databases as described by Plog et al. (2009). NCBI or Ensembl identifiers for CLCA2 sequences used are listed in Fig. S1. Exon-intron boundaries were established using WebScipio (Hatje, Hammesfahr & Kollmar, 2013) and aligned by GenePainter (Hammesfahr et al., 2013). Predicted protein domains were identified by the NCBI Conserved Domain Database (Lu et al., 2020), EMBL-EBI HMMER web server (Potter et al., 2018), Phobius webserver (Käll, Krogh & Sonnhammer, 2007), SOSUI (Hirokawa, Boon-Chieng & Mitaku, 1998), SignalP 3.0 (Bendtsen et al., 2004) algorithms and manual alignments. Asparagine (N)-linked glycosylation sites were predicted using the NetNGlyc webserver 1.0 (http://www.cbs.dtu.dk/services/NetNGlyc/). Turkey CLCA2 was not incorporated in the *in silico* analysis due to the low quality of the full-length gene and aa sequences stored in the NCBI and Ensemble databases (XP_031410715.1, XM_031554855.1, ENSMGAT00000009704.2). Phylogenetic relationship based on protein sequences of galline, quail, ostrich, feline, porcine and murine CLCA2 sequences was inferred by using the Maximum Likelihood method and JTT matrix-based model conducted in the MEGA X software package with 100 bootstrap replicates (Tamura, Stecher & Kumar, 2021) (S6).

Anti-gCLCA2 antibodies were generated similar to anti-porcine CLCA1 antibodies (Plog et al., 2009). In brief, an oligopeptide corresponding to aa 875 to 888 (WTAPGDDFDKGQAA) in the C-terminal region of gCLCA2 was synthesized and conjugated with *Limulus polyphemus* hemocyanin (LPH). The LPH- conjugated peptide was used for immunization of two rabbits. Specific IgG-antibodies were isolated from the antisera using a cyanogen bromide immunization-peptide coupled sepharose column and named gC2.

### Animals and tissues

In accordance with the 3R principle for the reduction of animal experiments, all tissues used in this study were obtained from the veterinary diagnostic pathology tissue archive of the Department of Veterinary Pathology, Freie Universität Berlin, Germany (VetPathFU) and originated either from the veterinary clinical diagnostic service unit or previous experimental studies. No animal was bred, raised, kept or euthanized specifically for this study. For chickens, 45 freshly frozen or formalin fixed and paraffin embedded (FFPE) tissues (Table S2) from ten-week old female individuals (*Gallus gallus domesticus*, Hampshire x White Leghorn, *n* = 3) and the gonads of age-matched male chickens (Hampshire x White Leghorn, *n* = 3) were used from (Bartenschlager et al., 2022). In brief, the tissues were by-products from slaughtered animals intended for human consumption. The animals had been bred, housed, and slaughtered in the Albrecht Daniel Thaer-Institute of Agricultural and Horticultural Sciences of the Humboldt-Universität zu Berlin, Germany under the permission of the State Office of Health and Social Affairs (approval number IC 114-ZH70). Weight at harvest was 1–1.2 kg (females) and 1.3–1.5 kg (males). The animals were raised in groups of 25, with infrared heat lamps offered until week five. They were fed with fledgling rearing feed until week eight and young hen feed afterwards. Miscanthus litter was used for housing enrichment. Harvesting was conducted according to national guidelines, which includes anesthesia by head blow and rapid exsanguination *via* jugular veins and carotid arteries. Females were harvested in the morning and males in the morning of the following day. FFPE tissues for immunohistochemical analyses, including esophagus and skin from shanks, abdomen and foot from one female and two male ostriches (*Struthio camelus*), three male quails (*Coturnix sp.*), and skin from three turkeys (*Meleagris gallopavo*) and skin of cats (*Felis catus*) were provided by the veterinary clinical postmortem diagnostic service unit of VetPathFU with no association to animal experiments. The tissues were obtained from the routine diagnostic spectrum to determine the cause of death of animals kept by private owners and free of histopathological changes. The permission to further use these tissues for research purposes was given by signature on the necropsy submission form by the owners. Additionally, FFPE skin samples from each of three mice and pigs from previous experimental studies (Braun et al., 2010; Plog et al., 2012a approval numbers T 0104/06 and G 0323/06, respectively) were obtained from the archive of VetPathFU and used for immunohistochemical analyses. In brief, 10-weeks-old female C57BL/6J mice were kept in cages enriched with nesting material. All animals had unlimited access to standard pelleted food and tap water. The room temperature was at 22 ± 2 °C and the relative humidity at 45–65%. A 12-h light/dark cycle was maintained. For experimental procedures, all mice were sacrificed by cervical dislocation in accordance with the national guidelines. Furthermore, the piglets (Euroc  × Piétrain) were 18 days old, male, castrated and kept for four weeks in flatdeck compartments in groups of six piglets enriched with playthings. All animals had unlimited access to mash food and tap water. The room temperature was 28 °C at stabling and was gradually decreased to 22 °C within 10 days with air humidity at approx. 65%. The light programme consisted of a 16 h light and 8 h dark phase. With 45 days of age, all piglets were anesthesized with ketamine hydrochloride (Ursotamin^®^, 10%; Serumwerk Bernburg AG, Germany) and azaperone (Stresnil^®^, Jansen-Cilag, Neuss, Germany) and euthanized using tetracaine hydrochloride, mebezonium iodide and embutramide (T61^®^, Intervet, Germany). All efforts were made to minimize animal discomfort and suffering.

### Molecular cloning and sequencing of *gCLCA2*

The *gCLCA2* open reading frame (ORF) was cloned as described with minor modifications (Bartenschlager et al., 2022). In brief, the *gCLCA2* ORF was amplified from a batch of tissues including pharynx, crop, proctodeum and footpad from animal #2 (Table S2). The gCLCA2 ORF was tagged with the enhanced yellow fluorescent protein (*EYFP*) at the C-terminus by cloning it into the p*EYFP*-N1 vector (Clontech, Mountain View, California, USA). The resulting plasmid (*gCLCA2#2*) was sequenced using the primer walking method (Data S5). Three plasmids from independent experiments yielded identical results.

### RT-qPCR tissue localization of *gCLCA2* mRNA

mRNA expression was analyzed using RT-qPCR as described (Bartenschlager et al., 2022). In brief, total RNA was isolated from galline tissues (Table S2), reverse transcribed, and the cDNA diluted to a final concentration of 1 ng/µl. Specific exon 13/14-boundaries spanning primers (upstream: 5′-CCAGGCTAACAGGACTACC-3′; downstream: 5′-GAAACCTCCTCTTCTGACCTGAAC-3′) were used to detect *gCLCA2* or the reference gene phosphoglycerate kinase (*PGK1*, upstream: 5′-AAAGTTCAGGATAAGATCCAGCTG-3′; downstream 5′-GCCATCAGGTCCTTGACAAT-3′; Olias et al., 2014) using a SYBR green qPCR assay (Thermo Fisher Scientific, Waltham, MA, USA). The *gCLCA2*-PCR product corresponds to the gCLCA2 protein region from aa760 to aa809 (QANRTTVPQTAMPWSHAMYIPGYVENGKLKMNPSRPPAIENNVQVRRGGF). *gCLCA2* mRNA was considered to be expressed when C_t_-values of 35 or less were detected in at least two out of three tested animals.

### Transient transfection of HEK293 cells

HEK293 cells (ATCC, Manassas, Virginia, USA) were transiently transfected as described with minor modifications (Bartenschlager et al., 2022). In brief, cells were grown in six-well plates in Dulbecco’s Modified Eagle’s Medium (DMEM) supplemented with 10% heat-inactivated fetal calf serum (FCS), 1% HEPES, and 1% penicillin/streptomycin. When reaching 80–90% confluence, the cells were transfected with 2 µg of a plasmid containing gCLCA2#2 or EYFP alone (mock) using 8 µl polyethylenimine (PEI) per well. 12 h post transfection, the cells were washed with phosphate buffered saline (PBS) and serum-free DMEM was added. 48 h after transfection, the cells of each well were lyzed using 500 µl radioimmunoprecipitation assay (RIPA) buffer supplemented with a protease inhibitor cocktail (complete Mini, EDTA-free, Roche Diagnostics, Rotkreuz, Switzerland). The protein concentration of supernatants and cell lysates were quantified using the bicinchoninic acid (Thermo Fisher Scientific, Waltham, Massachusetts, USA) method prior to freezing at −20 °C.

### Endoglycosidase treatment

For glycosylation analysis, lysates from *gCLCA2*-transfected cells were deglycosylated by incubation with 25 U/ml endo H, 50 U/ml PNGase F or left untreated at 37 °C over night according to the manufacturer’s protocols (New England Biolabs, Ipswich, Massachusetts, USA).

### Immunoblotting

Cell lysates and supernatants of *gCLCA2* transfected cells were analyzed using immunoblotting as described with minor modifications (Bartenschlager et al., 2022). In brief, samples of cell lysates or concentrated cell culture supernatant were reduced in 1,4-dithiothreitol (DTT) and separated using a 10% SDS-polyacrylamide gel electrophoresis. Proteins were transferred to a (PVDF)-membrane (https://www.linguee.de/englisch-deutsch/uebersetzung/polyvinylidene+fluoride.html) and blocked with 5% non-fat milk. Membranes were probed with antibody gC2 in a three-fold dilution series from 5 µg/ml to 0.05 µg/ml, or mouse monoclonal anti-YFP (cat. G163; ABM, Vancouver, Canada) diluted at 1:500, or mouse monoclonal anti-beta-actin (A5441, Sigma-Aldrich, St. Louis, Missouri, USA) diluted at 1:1,000. Membranes were incubated with horseradish peroxidase-conjugated goat anti-rabbit (115-035-068, Jackson Immuno Research Laboratories, Inc., West Grove, Pennsylvania, USA) or goat anti-mouse (111-035-144, Jackson Immuno Research Laboratories, Inc.) secondary antibodies and developed using enhanced chemiluminescence (Supersignal West Pico Plus, Thermo Fisher Scientific, Waltham, MA, USA). The gCLCA2 protein was only detected by this technique when using the anti-YFP antibody; however, it was undetectable when the gC2 antibodies were used.

### Immunocytochemistry of transfected HEK293 cells

Immunocytochemistry was performed as described with minor modifications (Bartenschlager et al., 2022). In brief, HEK293 (ATCC) cells were grown on 8-well tissue chamber slides and transfected with *gCLCA2#2* or *EYFP-* mock plasmids. 48 h after transfection, the cells were briefly fixed in ice-cold methanol followed by a 4% paraformaldehyde fixation for 10 min. After permeabilization with 0.1% Triton X-100 in PBS and blocking with 10% goat normal serum (GS) and 0.05% Tween 20 in PBS, cells were probed with untreated or pre-absorbed antibody gC2 each used at 2 µg/ml or irrelevant affinity-purified rabbit polyclonal anti-porcine CFTR antibody (Plog et al., 2010) (S3). Alexa fluor 568 conjugated goat anti-rabbit (AB_143157, Invitrogen, Carlsbad, California, USA) were used as secondary antibodies followed by 4′, 6-diamidino-2-phenylindole (DAPI) nuclear counterstain. All *in vitro* experiments were repeated three times.

### Tissue and cellular localization of gCLCA2 protein using immunohistochemistry and immunofluorescence

All galline tissues in which g*CLCA2* mRNA was detected at C_t_-values below 35 were analyzed *via* immunofluorescence to identify gCLCA2 expressing cell types. Furthermore, skin and esophagus from chicken, ostriches, quails, as well as skin of turkeys, mice, pigs, and cats were analyzed *via* immunohistochemistry. Immunofluorescence and immunohistochemistry were performed as described with minor modifications (Bartenschlager et al., 2022). In brief, FFPE-tissues were cut, mounted on adhesive glass slides, and dewaxed. For immunohistochemistry, endogenous peroxidase was blocked by adding 0.5% H_2_O_2_ in methanol. For immunofluorescence analysis, tissue sections of chickens were permeabilized with 0.1% Triton X-100 in PBS. Antigen was retrieved using 1 mg/ml recombinant protease from *Streptomyces griseus*. Slides were blocked with 10% Roti-ImmunoBlock and 20% GS in PBS for immunohistochemistry and 10% GS and 0.05% Tween 20 in PBS for immunofluorescence, both for 30 min. The slides were probed with the immunopurified gC2 or irrelevant affinity-purified rabbit polyclonal (anti-porcine CFTR, Plog et al., 2010) antibodies at 2 µg/ml. Additionally, mouse monoclonal anti-cytokeratin (AE1/AE3, M3515, Agilent Dako, Santa Clara, California, USA) antibodies were used at 1:400. For immunofluorescence, Alexa fluor 568-conjugated goat anti-rabbit (AB_143157; Invitrogen, Waltham, MA, USA) secondary antibodies were used diluted at 1:200, followed by DAPI nuclear counterstain. For immunohistochemistry with AE1/AE3 primary antibodies, 3,3′-diaminobenzidine (DAB) was added after incubation with goat anti-mouse biotinylated secondary antibodies (BA-9200; Vector Laboratories, Burlingame, California, USA) diluted at 1: 200 and an avidin-biotin complex. For immunohistochemistry with the gC2 primary antibody, DAB was added after incubation with the Histofine Simple Stain Mouse MAX PO anti-rabbit polymer kit (414341F; Nichirei Biosciences Inc., Tokyo, Japan). Potential cross reactivity of the gC2 antibody with pig, cat, mouse, turkey, quail and ostrich CLCA2 orthologues was tested by epitope sequence alignment using the NCBI Protein Blast (https://blast.ncbi.nlm.nih.gov/Blast.cgi?PAGE=Proteins, Table S4).

## Results

### Avian and mammalian *CLCA2* genes and their overall protein structures are conserved

**Figure 1:**
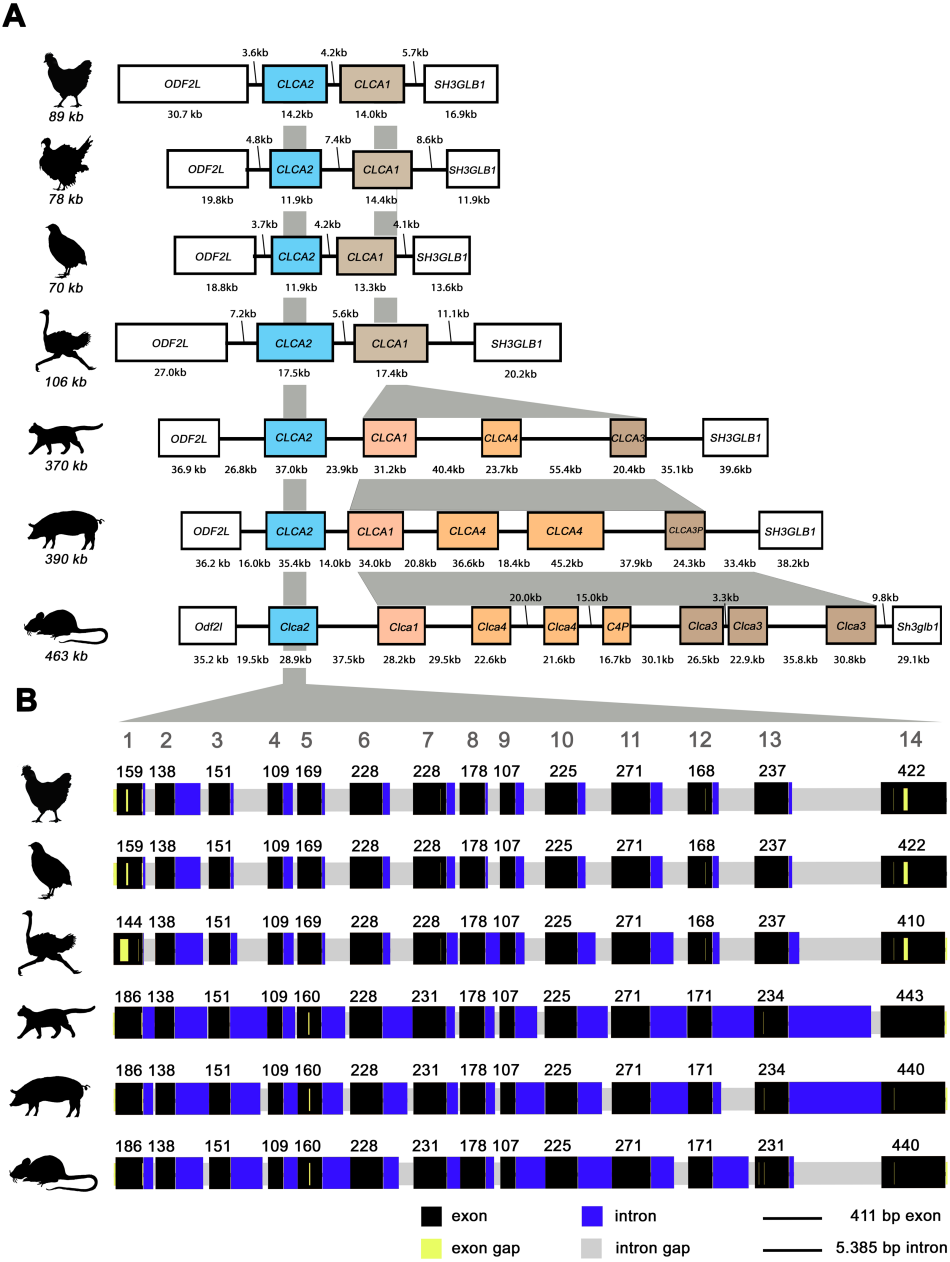
The genomic architecture of avian and mammalian *CLCA2* genes is highly conserved while *CLCA1* homologues are diverse only in mammals but not in birds. (A) Boxes indicate genes, black lines illustrate intergenic regions. Box colors highlight *CLCA* orthologues with white boxes indicating neighboring genes. Gray areas indicate regions of high sequence homology. The avian *CLCA* loci are scaled 2-fold larger for illustrative purposes. kb, kilobases. (B) The exon architecture of avian and mammalian *CLCA2* is highly conserved. Identical to mammals, a*CLCA2* genes are encoded by 14 exons with concurring exon lengths in exons 2, 3, 4, 6, 8, 9, 10 and 11. Black boxes represent exons and blue boxes depict introns. Gaps in the alignment are highlighted in yellow for exons and in grey for introns. Large grey digits at the top indicate exon numbers, small black digits indicate base pairs per exon. bp: base pairs.

**Figure 2:**
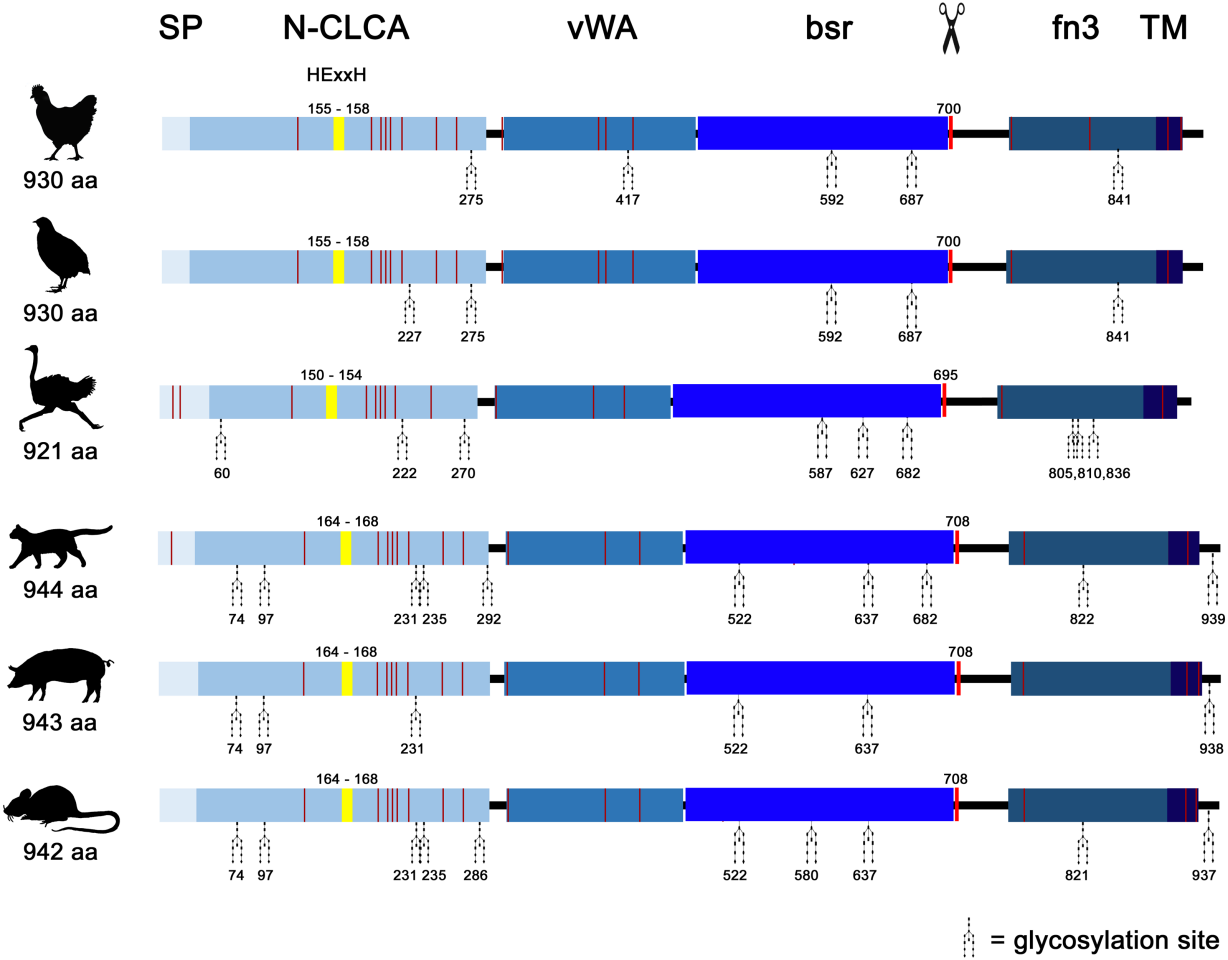
Avian and mammalian CLCA2 proteins share the canonical CLCA2 predicted protein architecture. Graphical alignment of CLCA2 orthologues. Protein domains are indicated by boxes in shades of blue and entitled by: SP, signal peptide; N-CLCA, N-CLCA domain; vWA, von Willebrand factor type A domain; bsr, beta sheet rich domain; fn3, fibronectin type III domain; TM, transmembrane domain. Yellow vertical boxes illustrate HExxH-motifs, red vertical boxes and the scissor sketch depict putative cleavage sites, vertical red lines constitute cysteines. Numbers indicate aa positions or lengths, respectively.

Similar to mammals (Bartenschlager et al., 2022; Mundhenk et al., 2018), avian *CLCA2* (a*CLCA2*) are single-copy genes located directly adjacent to the *ODF2L* gene in all species analyzed here (Fig. 1A). While in mammals the region between *CLCA2* and *SH3GLB1* comprise the complex and divergent *CLCA1/3/4* locus, birds contain only one single *CLCA1* gene (Fig. 1A). The presence of only two *CLCA* homologues but also the shorter intronic and intergenic regions that correspond to the more compact organization of avian genomes (Ellegren, 2005) make the consensus avian *CLCA* gene locus much smaller than that in mammals (Fig. 1A, Bartenschlager et al., 2022). The a*CLCA2* genes comprise 14 exons that encode for a single, putatively functional protein in all bird species analyzed here (Figs. 1B, 2), identical to all mammals investigated to date (Patel, Brett & Holtzman, 2009). The nucleotide numbers vary in only four exons by three to nine nucleotides between avian and mammalian *CLCA2* genes (Fig. 1B), causing only slightly distinct protein lengths (Fig. 2). Overall, our findings establish a high degree of evolutionary conservation of the genomic *CLCA2* architecture across birds and mammals (Fig. 1B). For all *CLCA2* orthologous genes investigated here, the predicted proteins share the canonical CLCA protein domain architecture as described (Patel, Brett & Holtzman, 2009). *In silico* prediction suggests a signal peptide within the first 22 to 43 aa, indicating entry into the secretory pathway which is a highly conserved trait in all avian and mammalian CLCA2 sequences (Fig. 2). An N-CLCA domain (PFAM identifier: pfam08434) containing a proteolytic HExxH-motif and a cysteine-rich domain is prepended to a vWA domain (PFAM identifier: pfam13519) and a bsr domain (Fig. 2, S1, Patel, Brett & Holtzman, 2009). In contrast to other CLCA proteins but consistent with maCLCA2, the vWA domain of CLCA2 of birds does not contain an intact metal ion dependent adhesion site (MIDAS, DxSxS-T4-D5). In concordance to maCLCA2, an fn3 (PFAM identifier: PF00041) and a TM domain are predicted in the C-terminal cleavage product for aCLCA2 (Fig. 2, Fig. S1). The presence of designated beta4-integrin binding motif (IBM, consensus sequence F(S/N)R(I/L/V)(S/T)S, Abdel-Ghany et al., 2003) appears less consistent. Human and porcine CLCA2 show such an IBM within the vWA domain (human aa480-485: FSRISS (Patel, Brett & Holtzman, 2009), pig aa480-485: FSRISS, Fig. S1) while it is absent from chicken, quail, ostrich, feline and murine CLCA2 (Fig. S1). Another IBM motif is lacking in the C-terminal cleavage product of birds and pigs whereas such a motif was found in human, feline and murine CLCA2 (human aa480-485: FSRISS (Patel, Brett & Holtzman, 2009), cat aa741-746: FSRVSS, Fig. S1, mouse aa740-745: FSRVSS (Patel, Brett & Holtzman, 2009), Fig. S1).

Noteworthy, aCLCA2 amino acid (aa) sequences are 921 to 930 aa long and therefore shorter than all of their mammalian homologues with 942 to 944 aa (Fig. 2). In addition, aCLCA2 do not contain a predicted glycosylation site after the predicted transmembrane domain (Fig. 2).

A phylogeny based on galline, quail, ostrich, feline, porcine and murine CLCA2 protein sequences revealed a monophyletic aCLCA2 group separate from maCLCA2 (Fig. S6).

### The gCLCA2 protein shares many biochemical properties with mammalian CLCA2 proteins

#### Posttranslational cleavage

**Figure 3:**
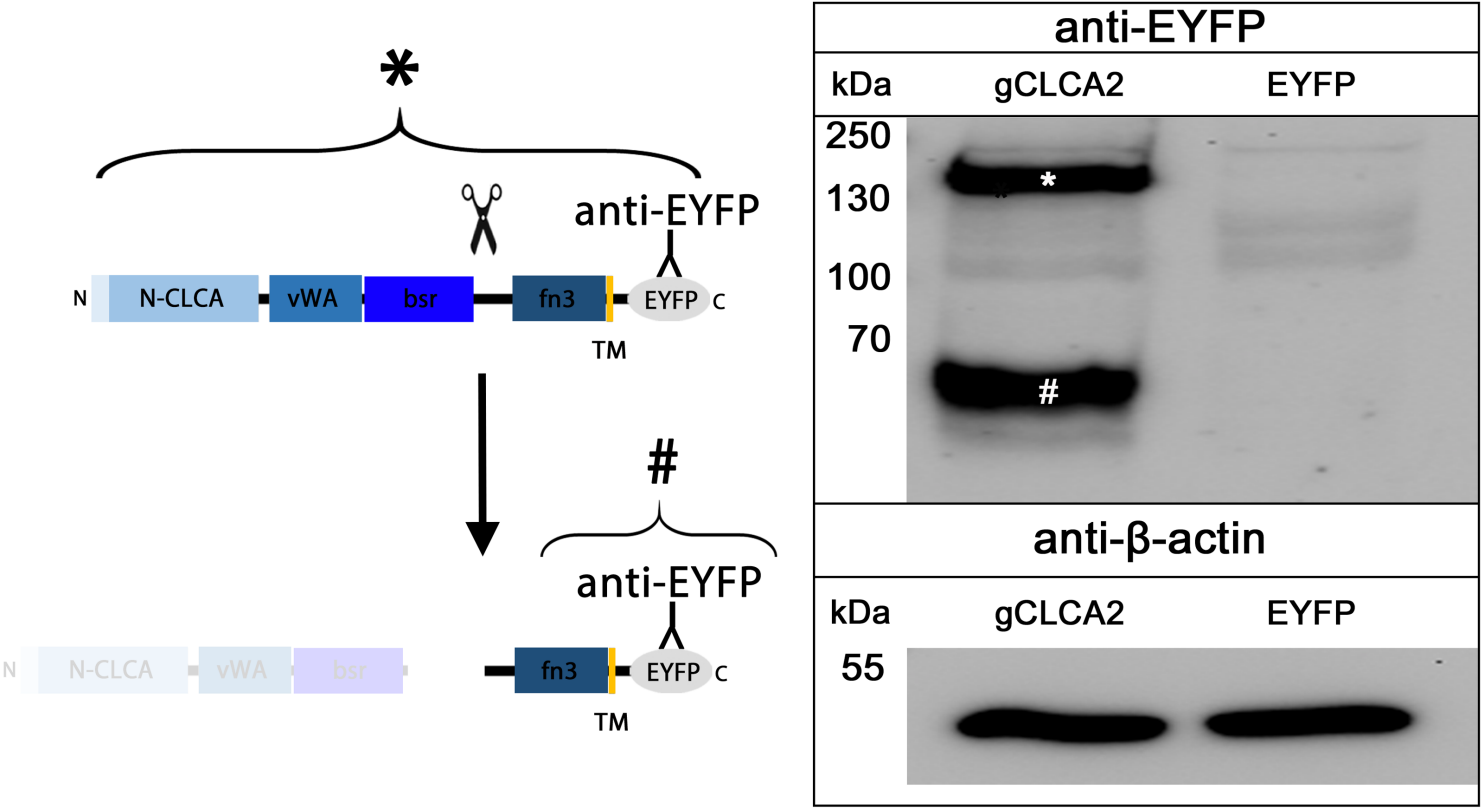
Posttranslational cleavage of the gCLCA2 precursor protein. Left panel: illustration of protein cleavage: N-CLCA: N-CLCA domain, vWA: von Willebrand factor type A domain, bsr, beta sheet rich domain; fn3, fibronectin type III domain; TM, transmembrane domain; EYFP, enhanced yellow fluorescent protein tag; scissor sketch, putative cleavage site; Y, antibody binding site. Right panel: immunoblot analysis of lysates from *gCLCA2* or *EYFP*-mock plasmid transfected HEK293 cells using an anti-EYFP antibody. An asterisk (*) indicates uncleaved precursor protein, a hash (#) indicates C-terminal cleavage product. To control for equal total protein loading, the samples were identically immunoblotted with primary anti-beta-actin antibodies (bottom panel). Representative image of three independent experiments are shown.

The cleavage of a precursor protein into a larger N- and a shorter C-terminal subunit belongs to the conserved properties of all maCLCA proteins (Patel, Brett & Holtzman, 2009). It is thought to be mediated by the zinc-binding HExxH motif in the N-CLCA domain, cleaving the protein at a canonical cleavage site (Bothe et al., 2012; Lenart et al., 2013; Pawłowski et al., 2006; Yurtsever et al., 2012). Consistently, canonical HExxH motifs and putative proteolytic cleavage sites were identified in aCLCA2 proteins (Fig. 2). The predicted cleavage was verified *in vitro* for gCLCA2 as a putative avian prototype by immunoblot analysis of lysates from heterologously transfected HEK293 cells. A band consistent with a precursor protein at approx. 145 kilodalton (kDa) and a band consistent with the C-terminal cleavage product of approx. 57 kDa were detected (Fig. 3), suggesting that posttranslational cleavage of CLCA2 also occurs in chicken.

### N-glycosylation and cleavage in the medial Golgi

**Figure 4:**
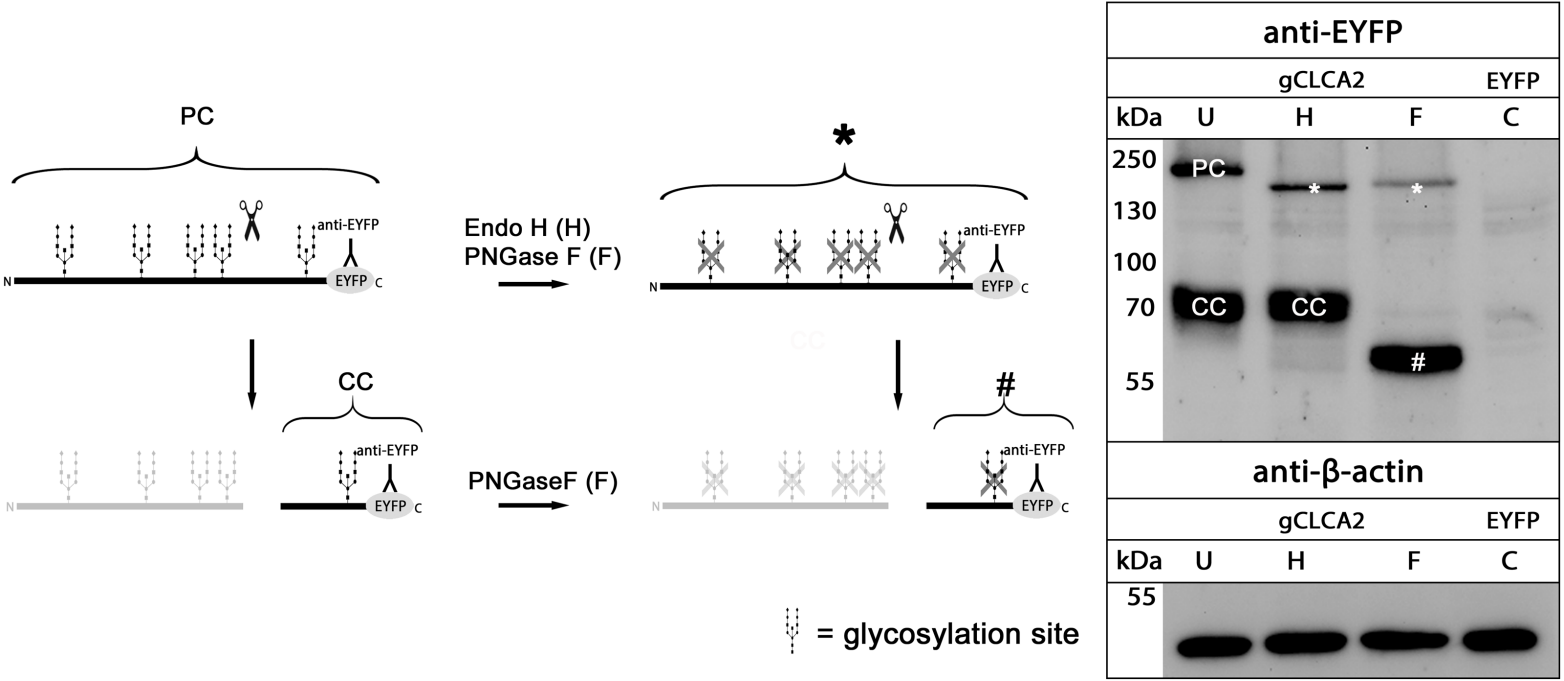
gCLCA2 is a multiple N-linked glycosylated protein and cleaved early in the medial golgi. Left panel: illustration of protein cleavage and deglycosylation: scissor sketch, cleavage; Y, antibody binding site; PC, precursor protein; CC, C-terminal cleavage product; asterisk, deglycosylated, uncleaved precursor protein; hash, deglycosylated C-terminal cleavage product. Right panel: immunoblot analysis of lysates from HEK293 cells transfected with *gCLCA2* or the *EYFP*-mock plasmids (C), using an anti-EYFP antibody. Lysates were treated with endoglycosidases endo H (H), PNGase F (F) or left untreated (U). To control for equal total protein loading, the samples were immunoblotted with anti-beta-actin antibodies (bottom panel). Representative image of three independent experiments.

maCLCA2 proteins are multiple N-linked glycosylated (Braun et al., 2010; Elble et al., 2006; Gruber et al., 1999; Plog et al., 2012b). Consistently, our *in silico* analyses predicted five N-linked glycosylation sites for aCLCA2 (Fig. 2). To verify this prediction experimentally, lysates from *gCLCA2-* transfected HEK293 cells were treated with endoglycosidases endo H and PNGase F and immunoblotted to identify the kind and extent of glycosylation. The approx. 145 kDa precursor protein was sensitive to endo H and PNGase F treatments (Fig. 4), resulting in a size shift from approx. 145 kDa to approx. 130 kDa, suggestive of an immature high mannose-type glycosylation pattern. In contrast, the C-terminal cleavage product was resistant to endo H treatment but sensitive to PNGase F treatment, as suggested by a reduction in size from approx. 57 kDa to approx. 54 kDa (Fig. 4). Our results indicate that virtually all predicted sites might be glycosylated, presuming a molecular weight of approx. 3 kDa per glycosylation (Pult et al., 2011). Furthermore, the complex high mannose-rich glycosylation pattern of the C-terminal cleavage products, in contrast to the immature glycosylated precursor protein, suggests cleavage of gCLCA2 early in the medial Golgi, similar to what has been observed for the murine CLCA2 (Braun et al., 2010). Thus, glycosylation and cleavage in the medial Golgi also appear as conserved traits.

### Anchoring in the plasma membrane

**Figure 5:**
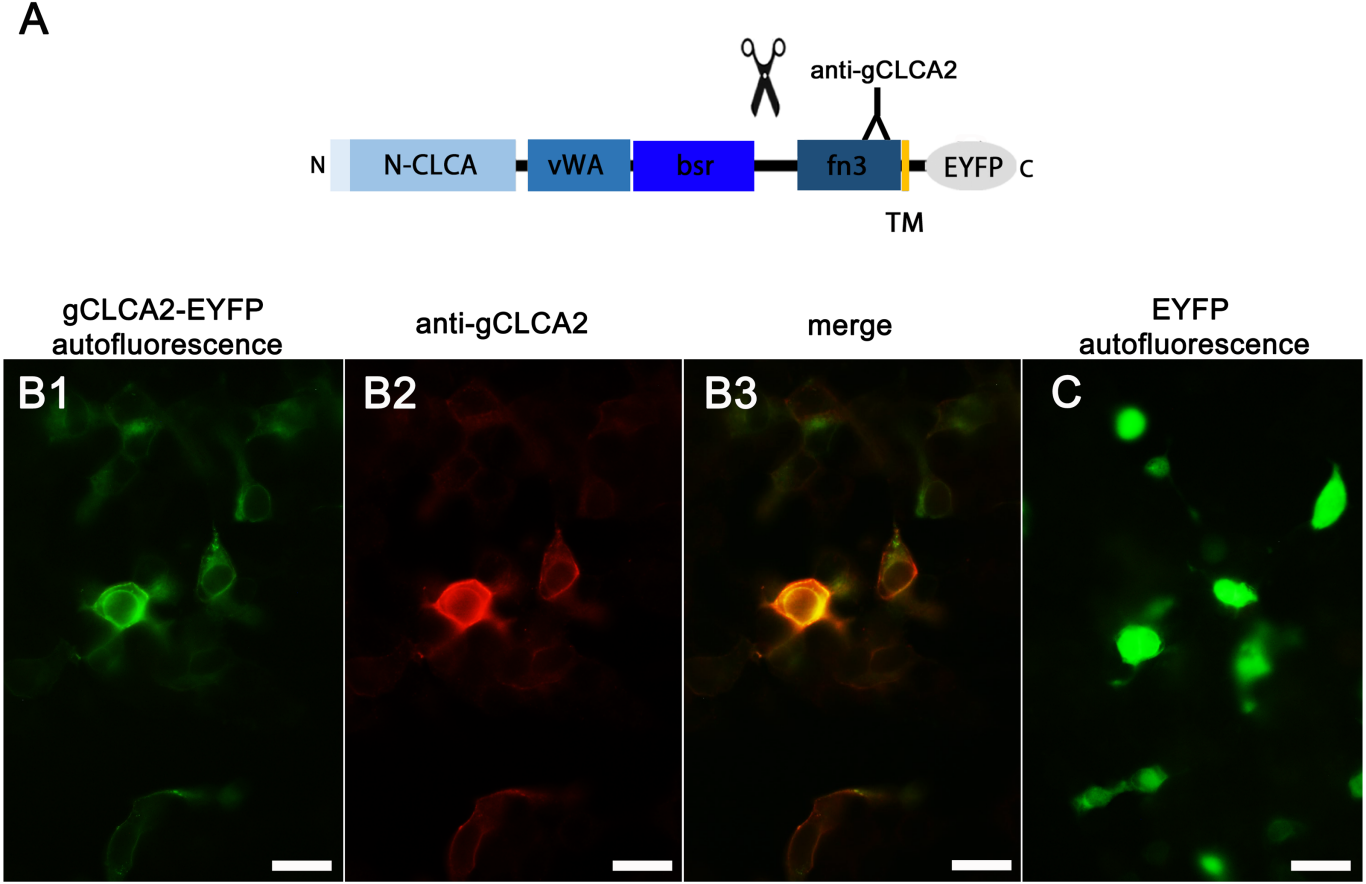
Heterologously expressed gCLCA2 is associated with the plasma membrane. (A) illustration of the anti-gCLCA2 binding site: N-CLCA, N-CLCA domain; vWA, von Willebrand factor type A domain; bsr, beta sheet rich domain; scissor sketch, putative cleavage site; fn3, fibronectin type III domain; TM, transmembrane domain; EYFP, enhanced yellow fluorescent protein tag; Y, antibody binding site. (B) auto- and immunofluorescent localization of the C-terminal cleavage product of gCLCA2 using antibody gC2 in HEK293 cells transfected with the *gCLCA2* plasmid. B1, green signal; EYFP, autofluorescence; B2, red signal anti-gCLCA2 immunofluorescence; B3, orange; merged signals of EYFP autofluorescence and anti-gCLCA2 immunofluorescence. Alexa fluor 568-conjugated secondary antibodies. (C) autofluorescence (green signals) of HEK293 cells transfected with the *EYFP* mock plasmid. Bars indicate 20 µm. Representative images of three independent experiments are shown.

Identically to its maCLCA2 orthologues, a TM domain in the C-terminal subunit was predicted for aCLCA2, which anchors the protein to the plasma membrane (Fig. 2; Braun et al., 2010; Elble et al., 2006; Gruber et al., 1999; Plog et al., 2012b). Consistently, a prominent green autofluorescent EYFP signal was detected along the plasma membrane of HEK293 cells transfected with the *gCLCA2* construct that contained an EYFP tag downstream to the TM domain (Figs. 5A and Figs. 5B1). A coinciding signal (Fig. 5B3) was found by immunocytochemistry using the anti-gC2 antibody directed against the fn3 domain of the protein, which is located upstream to the C-terminal transmembrane domain (Fig. 5B2). This is in contrast to the diffuse green signal of cytosolic EYFP protein (Fig. 5C). Therefore, anchorage of the CLCA2 protein in the cell membrane *via* a TM domain seems also conserved with mammals.

### aCLCA2 is expressed in stratified squamous epithelia of skin and mucous membranes

**Figure 6:**
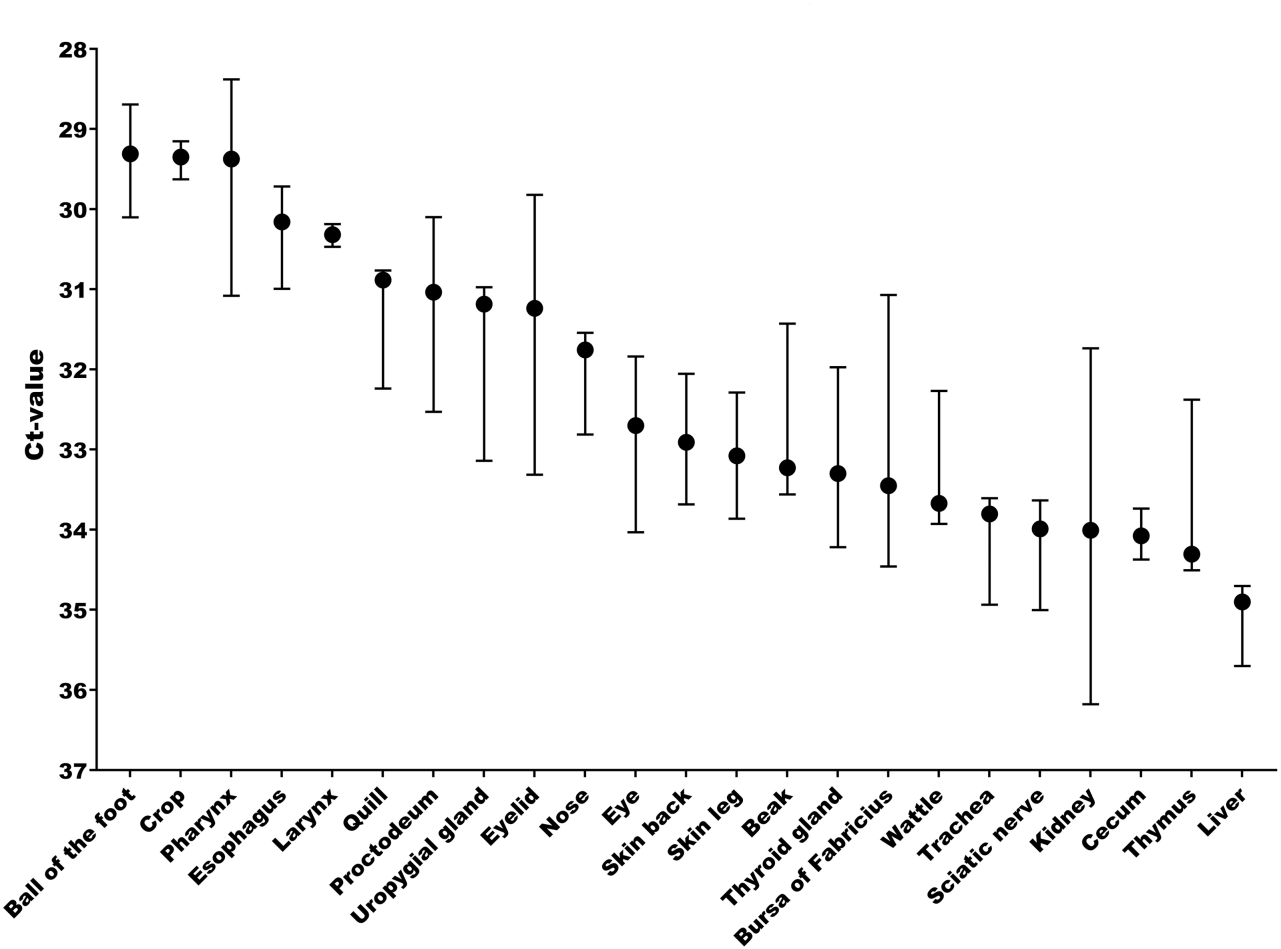
Abundant g*CLCA2* mRNA was detected by quantitative RT-PCR in tissues containing keratinizing epithelium. Black dots represent median Ct values, error bars indicate the range. *n* = 3 animals per group. Ct, cycle threshold.

**Figure 7:**
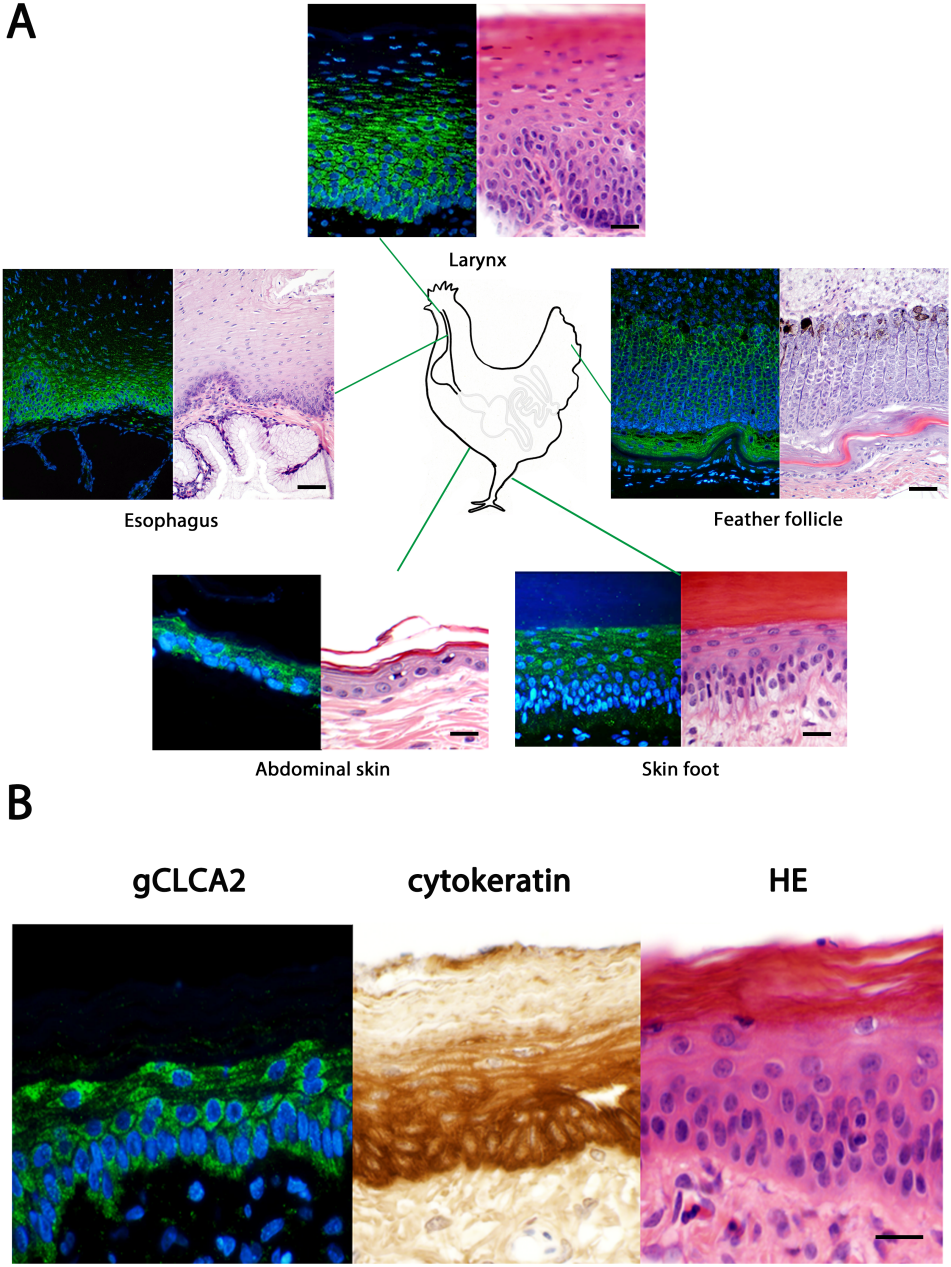
Immunofluorescence localization of the gCLCA2 protein in the cells of stratified/cornified epithelia of the skin, mucus membranes and feather follicles. (A) Immunofluorescence on galline tissues using the gC2 antibody (left panels), compared to hematoxylin and eosin (HE) stained tissue sections of FFPE-samples (right panels) for structural comparisons. Alexa fluor 488-conjugated secondary antibodies were used for visualization (green) and DAPI as nuclear counterstain (blue). Bars: 10 µm. (B) the gCLCA2 protein is located in stratified epithelial cells characterized by the expression of cytokeratins. Left panel: Immunofluorescent localization of the gCLCA2 protein in stratified epithelium of wattle skin using antibody gC2 and Alexa fluor 488-conjugated secondary antibodies with DAPI nuclear counterstain (blue). Central panel: immunohistochemical localization of acidic and basic cytokeratins in a serial tissue section using the AE1/AE3 antibody cocktail, biotinylated anti-mouse IgG secondary antibodies conjugated with horseradish peroxidase and DAB as chromogen (brown) with hematoxylin counterstain (blue). Right panel: tissue morphology visualized using hematoxylin (blue) and eosin (pink) stain on a consecutive tissue section. Bars: 10 µm. Representative images of sections from three animals.

**Figure 8:**
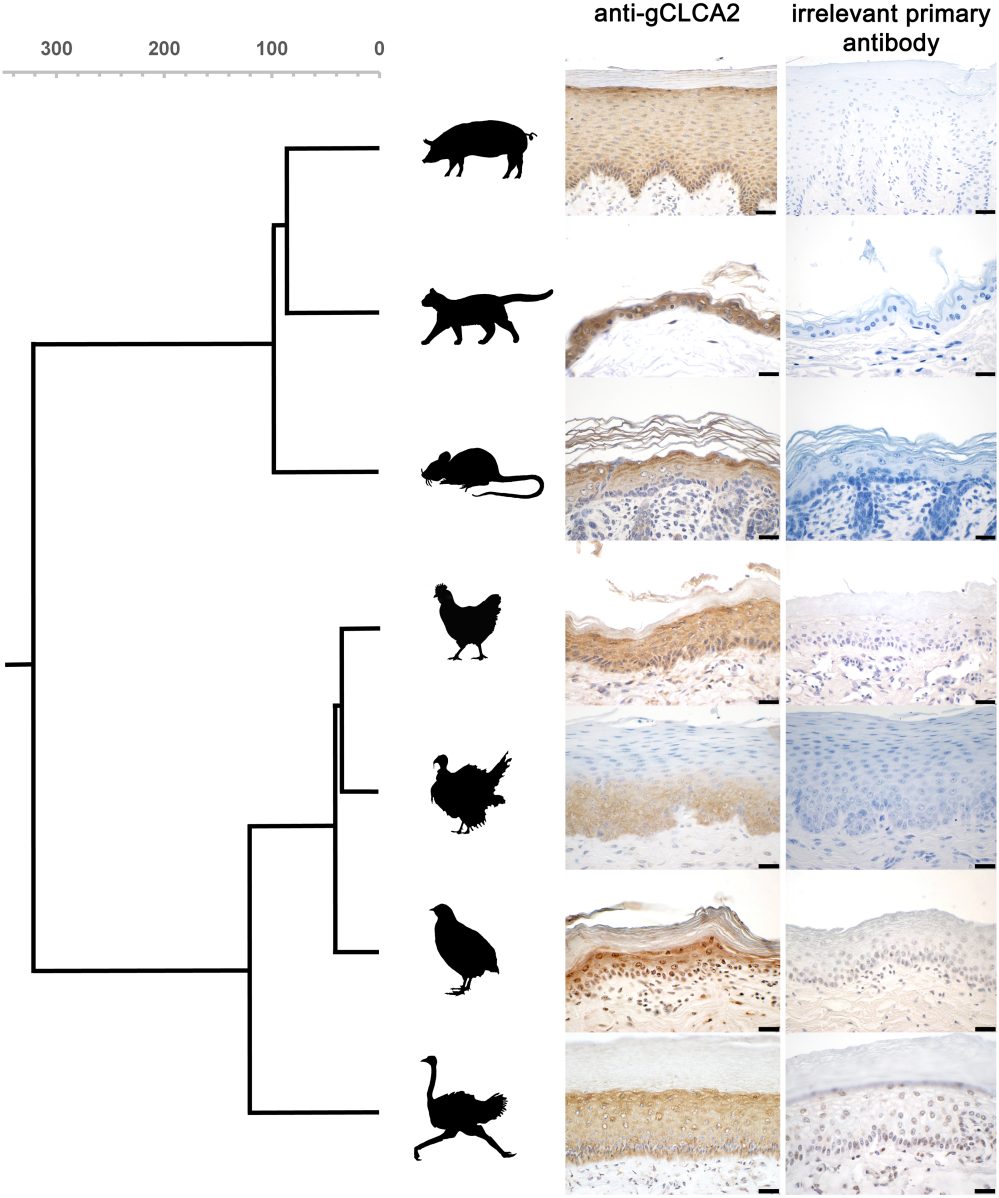
CLCA2 expression is conserved in viable epidermal keratinocytes across avian and mammalian species. Immunohistochemistry using the gC2 antibody and an irrelevant control antibody (anti-porcine CFTR). Biotinylated anti-mouse secondary antibodies and DAB as chromogen (brown) and hematoxylin counterstain (blue). Gray scalebar: species divergence in million years. Black bars: 20 µm. Representative images of sections from three animals per species.

The tissue and cellular expression patterns of gCLCA2 were examined at the mRNA- and protein-levels. *gCLCA2* mRNA was detected by RT-qPCR in all tested locations of the skin (back, foot, wattle, ball of the foot, proctodeum) and skin appendages (feather follicle, beak, uropygial gland) as well as in organs with keratinizing mucosal membranes, such as the nose, pharynx, esophagus, crop, and proctodeum (Fig. 6, Table S2). Additionally, *gCLCA2* mRNA was found in the trachea, cecum, kidney, bursa of Fabricius, thyroid gland, sciatic nerve, eye and in the liver (Fig. 6, Table S2). gCLCA2 protein was exclusively detected immunohistochemically in keratinocytes of the skin, skin appendages and keratinizing mucosal membranes of the larynx, esophagus and crop (Fig. 7A, Fig. S3). gCLCA2 was localized in all layers of the stratified epithelium similar to the epithelial cell marker cytokeratins (Fig. 7B). At the subcellular level, signals consistently appeared as multiple, evenly distributed dots within the cytosol, with no specific signal enrichment detected at the plasma membrane. However, we failed to detect the CLCA2 protein in other tissues with notable *gCLCA2* mRNA presence (Fig. 6). Abundant expression in epidermal keratinocytes appears as a consistent hallmark of CLCA2 proteins, as verified in chicken, turkeys, quails, ostriches, cats, pigs and mice (Fig. 8).

## Discussion

Previous investigations on the *CLCA* gene family have revealed unusually complex evolutionary developments in some of its members (Bartenschlager et al., 2022; Mundhenk et al., 2018). The divergent cluster of mammalian *CLCA1/3/4* genes is characterized by multiple and independent duplication and inactivation events. This suggests flexible adaptation to environmental conditions and separation and specification of gene functions (Mundhenk et al., 2018; Patel, Brett & Holtzman, 2009; Plog et al., 2015). In sharp contrast, the *CLCA2* gene appears as a consistent single intact gene in mammalian and avian species examined here (Fig. 1A). It thus appears that *CLCA2* is the most conserved gene of the family, providing an opportunity to gain a consistent and detailed insight into basic properties of *CLCA* genes. Here, our systematic comparisons of the *CLCA2* genomic and protein structures, biochemical properties and tissue as well as cellular expression levels confirmed the high degree of conservation within and between birds and mammals.

Similar to all previously studied *CLCA* genes, avian *CLCA2* (a*CLCA2)* genes are encoded by 14 exons with small in-frame deletions, compared to ma*CLCA2* (Fig. 1B), leading to slightly shorter protein sequences. Furthermore, aCLCA2 match the canonical protein architecture of CLCA that had been described for mammalian species (Braun et al., 2010; Elble et al., 2006; Evans, Thoreson & Beck, 2004; Patel, Brett & Holtzman, 2009; Plog et al., 2012b), namely the consecutive sequence of a signal peptide followed by the domains N-CLCA, vWA, bsr, fn3. These domains as well as the consistent proteolytic cleavage motif located between bsr and fn3 might constitute a not yet defined common functional property of intact CLCA gene family members. Obviously, however, the evolutionary turnover has modified functional properties, as suggested by the lack of an otherwise consistent TM domain in maCLCA1 (Bartenschlager et al., 2022). Similarly, an intact MIDAS site that is present in the mammalian CLCA1/3/4 cluster and its avian CLCA1 orthologue (Bartenschlager et al., 2022) was found to be interrupted in CLCA2 proteins in mammals and birds. This conserved feature of CLCA2 might point to a similar sub-functionalization in mammals and birds.

Cleavage of gCLCA2 into a larger N-terminal part and a smaller membrane-anchored C-terminal tail is presumably caused by proteolytic cleavage between the bsr and fn3 domain in the medial Golgi. This is similar to what has been found in its murine orthologue (Braun et al., 2010) whereas the human orthologue is cleaved extracellularly after its insertion into the plasma membrane (Elble et al., 2006). Thus, cleavage in the medial Golgi appears to be the evolutionarily older, prototypical process, which might have been changed in certain lineages, including humans.

The CLCA2 protein was consistently found to be expressed in epidermal keratinocytes of various cutaneous and mucosal body coverings of chicken, turkey, quail and ostrich. This highly select cellular protein expression is similar to that of human, murine, feline, and porcine orthologues (Braun et al., 2010; Connon et al., 2006; Connon et al., 2004; Erickson, Gruber & Mundhenk, 2020; Hamalainen et al., 2021; Plog et al., 2012b; Seltmann et al., 2018). Despite the obvious differences of avian and mammalian skin anatomy (Akat et al., 2022), CLCA2 expression in keratinizing epithelial cells seems to be highly conserved, supporting the notion that skin and skin appendages including hair in mammals and feathers in birds share a common ancestry (Di-Poï & Milinkovitch, 2016). The idea of the symplesiomorphic nature of CLCA2 protein abundance in hair (Plog et al., 2012b) and feather follicles might be extended to other sites as well. It had been reported that corneal epithelial cells of galline embryos express CLCA2 protein (Connon et al., 2006). Like in skin, hair and feathers, expression in corneal epithelial cells is plausible as they derive from a common ectodermal origin with keratinocytes. Accordingly, *gCLCA2* specific mRNA was found in the ten-week old chicken eye in our study, while no protein was detected using immunohistochemistry. It will be interesting to explore whether impaired sensitivity for protein detection was the reason for this discrepancy or if CLCA2 expression in corneal epithelial cells depends on the developmental status. Similarly, the detection of *gCLCA2* mRNA in several other tissues, albeit at lower mRNA expression levels, with concomitant lack of detectable CLCA2 protein is in line with similar findings on murine CLCA2 (Braun et al., 2010). On the one hand, the *gCLCA2* specific amplicon detected by RT-qPCR is slightly (129 nucleotides) upstream to the sequence that encodes the antibody-binding site for the anti-gCLCA2 antibody used in this study. Therefore, it cannot be fully excluded that these tissues, which apparently express *gCLCA2*-mRNA but lack expression of gCLCA2 protein, may express truncated or otherwise modified variants of *gCLCA2*. On the other hand, this discrepancy underscores established difficulties in exploring gene products that appear at low expression levels or are restricted to small niches of expression sites. Theoretically, non-translated transcripts of *CLCA2* might also have regulatory properties, but to our knowledge, no such mechanisms have ever been proposed for any member of the *CLCA* gene family. For this reason, we propose the most relevant role of CLCA2 in keratinocytes of skin, hair and feather follicles in mammals and birds, respectively. The highly conserved expression in the epidermis may suggest an indispensable role for CLCA2 in keratinocyte function during the course of evolution. It remains to be established, however, to which functional aspect this may pertain, including skin barrier function, osmolar homeostasis, cell signaling or local immunity.

Despite the fact that most of the assessed traits appear conserved between aCLCA2 and maCLCA2, two inconsistencies were found: first, while we failed to detect the gCLCA2 protein in the airways of chicken, it is abundantly expressed in epithelial cells of the respiratory tract of some albeit not all mammals (Dietert et al., 2015). Given the established evolutionary relationships, this may propose a potential adaptation of CLCA2 in the respiratory tract during mammalian evolution and its subsequent loss in cats or other carnivores. Theoretically, a more ancient function of CLCA2 in the respiratory tract and an independent loss in avian and select mammalian species may be conceived, possibly to be confirmed in a common ancestor of avian and mammals. Even more complicated to interpret is the presence of IBM motifs in the vWA and fn3 domains of CLCA2 that are thought to mediate cell–cell adhesion via interaction between CLCA2 and beta4-integrin (Abdel-Ghany et al., 2003; Abdel-Ghany et al., 2001). However, the inconsistency of functional data on this motif and its relatively loose consensus sequence F(S/N)R(I/L/V)(S/T)S raise doubt on whether this motif has any physiological function in CLCA2.

To date, knowledge about functional properties of CLCA2 are still limited, as is the case for CLCA proteins in general. CLCA2 seems to lack significant associations with human diseases, (OMIM Database entry #604003), which is usually the prime driver for generating animal models to investigate more complex functions *in vivo*. This is in contrast to ma *CLCA1*, for which its proposed role as modifier in cystic fibrosis (Hauber et al., 2003; Ritzka et al., 2004; Young et al., 2007) has stimulated the generation of several knockout models in mice (Erickson et al., 2018; Erickson et al., 2015; Long et al., 2006; Mundhenk et al., 2012; Nyström et al., 2018; Patel et al., 2006; Robichaud et al., 2005). The lack of a relevant phenotype in any of these models raises general reservations regarding the suitability of deleting a gene for exploring its function, but in the case of *CLCA*, the interpretation was complicated even further. The similar protein architectures of CLCAs and, at least in mice, the overlapping tissue expression patterns of CLCA1 and CLCA2 (Long et al., 2006; Mundhenk et al., 2012; Patel et al., 2006; Robichaud et al., 2005) immediately suggested a functional cross-compensation between different CLCA members in the respiratory tract. Given the tissue expression pattern of distinct CLCA members in the epidermis of mice (Seltmann et al., 2018), a mutually overlapping and possibly redundant function of certain CLCAs would also question the suitability of the mouse as model organism for studying *CLCA2* function keratinocytes. In chicken, however, the overall architecture of the *CLCA* locus is much less complex than that in mammals (Bartenschlager et al., 2022; Mundhenk et al., 2018). Moreover, the non-overlapping expression of *gCLCA1* in enterocytes (Bartenschlager et al., 2022) and *gCLCA2* in keratinocytes largely excludes the possibility of mutual compensation in chickens. In combination with the high degree of conservation among avian and mammalian *CLCA2* and the recent progress in genome editing techniques in birds (Chojnacka-Puchta & Sawicka, 2020; Morin, Véron & Marcelle, 2017), the simple structure of the galline *CLCA* gene locus together with its distinct expression pattern might provide a suitable setting for *in vivo* explorations of *CLCA2* functions using genetically edited chickens.

## Conclusions

Our data provide strong evidence for a high conservation of *CLCA2* in mammalian and avian species during evolution. This is in stark contrast to the dynamics and proposed complex functional adaptations in the *CLCA1/3/4* cluster. The slow evolutionary dynamics of *CLCA2* genes insinuates a significant degree of negative selection and a strong functional conservation of *CLCA2* orthologues among birds and mammals, particularly in epidermal keratinocytes. This proposes g*CLCA2* as a suitable object for studying basic functional properties of *CLCA* 2. Furthermore, the simple structure of the *CLCA* locus in birds and distinct and rather simple expression patterns in chicken may serve as an ideal frame to experimentally address overall CLCA functions in the latter, rather than in mammals.

##  Supplemental Information

10.7717/peerj.14202/supp-1 Supplemental Information 1 Multiple sequence alignment (MSA) of CLCA2 protein sequences from chicken, quail, ostrich, cat, pig and mouse Predicted signal peptide sequences were removed and sequences were aligned using MUSCLE algorithm implemented in MEGA X software package with default parameters. MSA was visualized using Jalview software and conserved amino acids were highlighted in blue using the threshold value of 30. N-CLCA, vWA, bsr and fn3 domains were annotated according to (Patel, Brett & Holtzman, 2009). The TM domains were defined based on SOSUI predictions and highlighted in yellow. Red line indicates putative cleavage site. Yellow box and yellow letters D4, T5 indicate the destroyed metal ion dependent adhesion site (MIDAS) site, green boxes indicate the intact beta4-integrin binding motif (IBM). NCBI or Ensembl identifiers of each sequence are listed on the left side.
Patel AC, Morton JD, Kim EY, Alevy Y, Swanson S, Tucker J, Huang G, Agapov E, Phillips TE, and Fuentes ME. 2006. Genetic segregation of airway disease traits despite redundancy of calcium-activated chloride channel family members. *Physiological genomics* 25:502-513.

10.7717/peerj.14202/supp-2 Supplemental Information 2 gCLCA2 and PGK1 qPCR raw data (Ct-values)

10.7717/peerj.14202/supp-3 Supplemental Information 3 Testing the gC2 antibody for specificity EYFP auto- and immunofluorescence of HEK293 cells transiently transfected with the *gCLCA2#2* plasmid (A, B, D, E, F) or *EYFP*-mock plasmid (EYFP, C) plasmids. (B) The signal (red) detected with the gC2 primary antibody was virtually identical to the autofluorescence signal (green) in (A). No specific signals were detected after incubation of *EYFP*-mock transfected cells with the antibody (C) or when *gCLCA2#2* transfected cells were incubated with an irrelevant antibody (anti-pCFTR, (Plog et al., 2010)) (D). The incubation of *gCLCA2#2* transfected cells with the pre-absorbed gC2 antibody using the specific peptide for immunization did not detect any gCLCA2 protein (E). In contrast, the pre-absorption of the gC2 antibody with an irrelevant peptide did not reduce the signal intensity (F). After incubation of FFPE sections from chicken skin with the gC2 antibody, a prominent green signal was identified throughout all layers of the epidermis (G). This signal was not detected when identical sections were incubated with an irrelevant (anti-pCFTR) primary antibody. Alexa fluor 568 (B–F) and 488 (G–H)-conjugated secondary antibodies with DAPI counterstain (blue, G–H). Bars indicate 20 µm. Exposure times were 1 s for A, 190 ms (ms) for B–H and 333 ms for G–H (green channel).

10.7717/peerj.14202/supp-4 Supplemental Information 4 In-silico prediction of gC2 antibody cross reactivity with quail, ostrich, turkey, pig, cat and mouse CLCA2 orthologues The comparison of the galline epitope, against the gC2 antibody was raised, with avian and porcine CLCA2 orthologues showed an expectation (e) value lower than the generally accepted threshold of 10^−5^ for possible cross-binding (McClain et al. 2017). Although the e values from feline and murine sequences were slightly above the threshold, convincing signals were detected in our study corresponding to the previous findings (Erickson, Gruber & Mundhenk, 2020), Braun et al. 2009, (Hamalainen et al., 2021).
+: two sequences are similar but not highly similar, -: aa gap.
McClain S. 2017. Bioinformatic screening and detection of allergen cross-reactive IgE-binding epitopes. *Molecular nutrition & food research* 61:1600676.
Erickson NA, Nyström EE, Mundhenk L, Arike L, Glauben R, Heimesaat MM, Fischer A, Bereswill S, Birchenough GM, and Gruber AD. 2015. The goblet cell protein Clca1 (alias mClca3 or Gob-5) is not required for intestinal mucus synthesis, structure and barrier function in naive or DSS-challenged mice. *PLOS ONE* 10:e0131991.
Braun J, Bothe MK, Mundhenk L, Beck CL, and Gruber AD. 2010. Murine mCLCA5 is expressed in granular layer keratinocytes of stratified epithelia. *Histochem Cell Biol* 133:285-299. 10.1007/s00418-009-0667-0
Hamalainen L, Bart G, Takabe P, Rauhala L, Deen A, Pasonen-Seppanen S, Karkkainen E, Karna R, Kumlin T, Tammi MI, and Tammi RH. 2021. The calcium-activated chloride channel-associated protein rCLCA2 is expressed throughout rat epidermis, facilitates apoptosis and is downmodulated by UVB.* Histochem Cell Biol*. 10.1007/s00418-021-01962-5.

10.7717/peerj.14202/supp-5 Supplemental Information 5 SNPs of the g*CLCA2* clone used in this study

10.7717/peerj.14202/supp-6 Supplemental Information 6 Evolutionary analysis of CLCA2 protein sequences from chicken, quail, ostrich, cat, pig and mouse by Maximum Likelihood method The evolutionary history was inferred by using the Maximum Likelihood method and JTT matrix-based model. The tree with the highest log likelihood (−6846.32) is shown. The percentage of trees in which the associated taxa clustered together is shown next to the branches. Initial tree(s) for the heuristic search were obtained automatically by applying Neighbor-Join and BioNJ algorithms to a matrix of pairwise distances estimated using the JTT model, and then selecting the topology with superior log likelihood value. A discrete Gamma distribution was used to model evolutionary rate differences among sites (5 categories (+G, parameter = 13.4417)). The rate variation model allowed for some sites to be evolutionarily invariable ([+I], 17.81% sites). This analysis involved 6 amino acid sequences. All positions containing gaps and missing data were eliminated (complete deletion option). Predicted signal peptide sequences were removed before analysis. There were a total of 873 positions in the final dataset. Evolutionary analyses were conducted in MEGA X.

10.7717/peerj.14202/supp-7 Supplemental Information 7 Original blots

## Additional Information and Declarations

### Data Availability

The following information was supplied regarding data availability: The raw data, the individual Ct values of gCLCA2 and PGK, a loading control, and the raw, uncropped blot images are available in the Supplementary Files.
