# Supplementary figures and images for: Genomic, biochemical and expressional properties reveal strong conservation of the CLCA2 gene in birds and mammals
Source: PeerJ. 2022 Nov 8;10:e14202. doi: 10.7717/peerj.14202 (PMC9651043; doi:10.7717/peerj.14202)

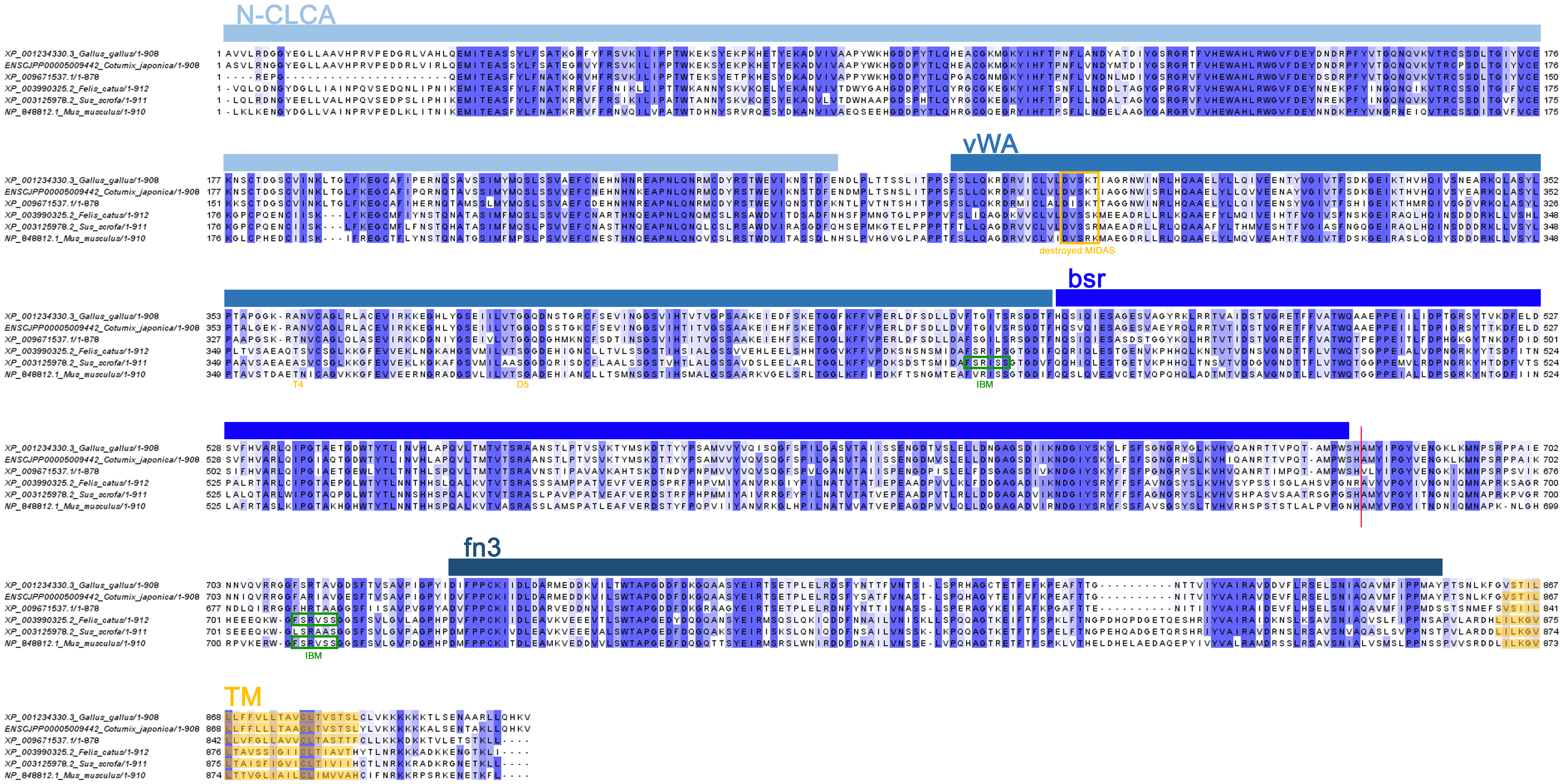

Supplement: Supplemental Information 1 — Predicted signal peptide sequences were removed and sequences were aligned using MUSCLE algorithm implemented in MEGA X software package with default parameters. MSA was visualized using Jalview software and conserved amino acids were highlighted in blue using the threshold value of 30. N-CLCA, vWA, bsr and fn3 domains were annotated according to (Patel, Brett & Holtzman, 2009). The TM domains were defined based on SOSUI predictions and highlighted in yellow. Red line indicates putative cleavage site. Yellow box and yellow letters D4, T5 indicate the destroyed metal ion dependent adhesion site (MIDAS) site, green boxes indicate the intact beta4-integrin binding motif (IBM). NCBI or Ensembl identifiers of each sequence are listed on the left side. Patel AC, Morton JD, Kim EY, Alevy Y, Swanson S, Tucker J, Huang G, Agapov E, Phillips TE, and Fuentes ME. 2006. Genetic segregation of airway disease traits despite redundancy of calcium-activated chloride channel family members. Physiological genomics 25:502-513. [file peerj-10-14202-s001.jpg]

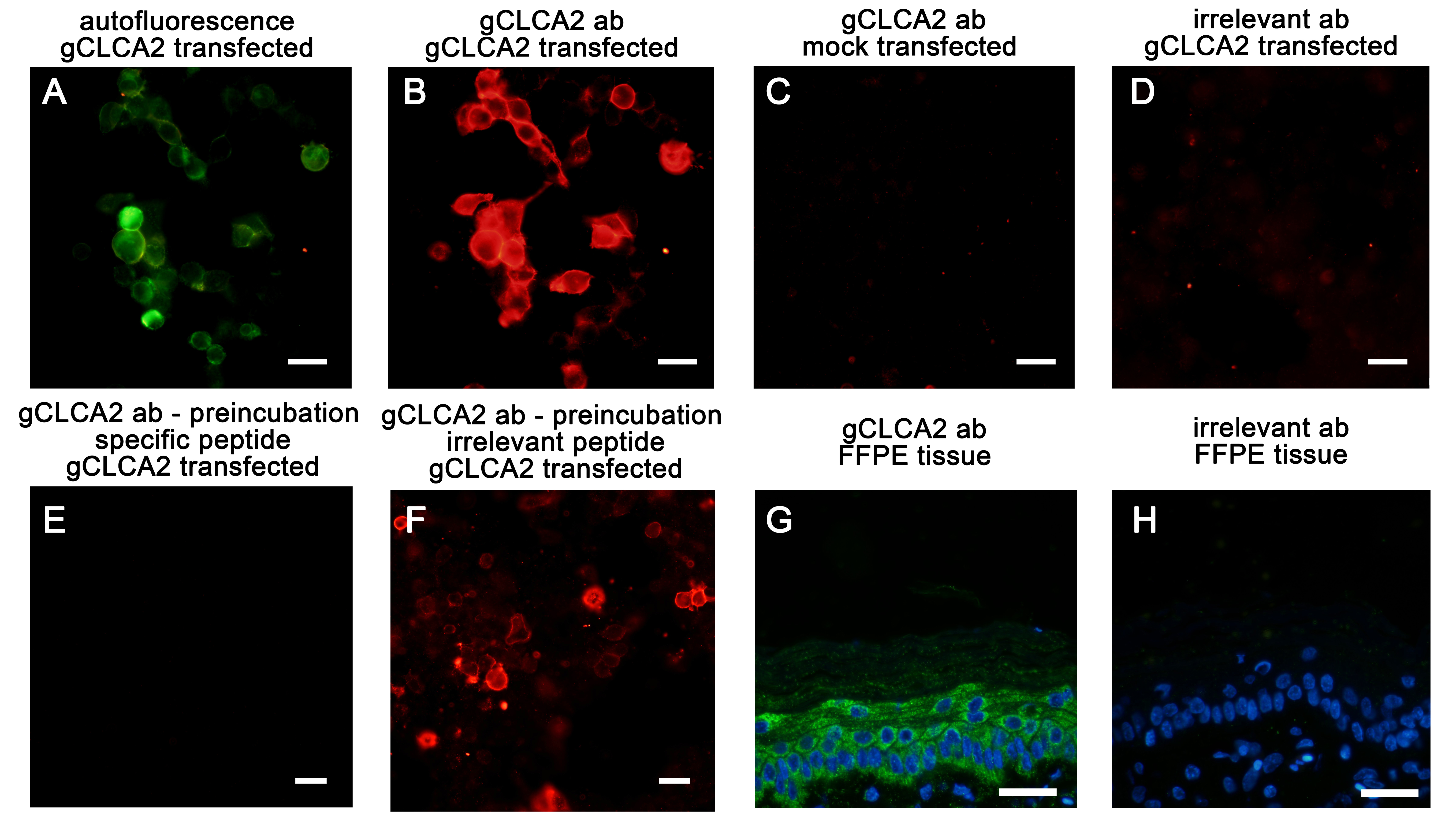

Supplement: Supplemental Information 3 — EYFP auto- and immunofluorescence of HEK293 cells transiently transfected with the gCLCA2#2 plasmid (A, B, D, E, F) or EYFP-mock plasmid (EYFP, C) plasmids. (B) The signal (red) detected with the gC2 primary antibody was virtually identical to the autofluorescence signal (green) in (A). No specific signals were detected after incubation of EYFP-mock transfected cells with the antibody (C) or when gCLCA2#2 transfected cells were incubated with an irrelevant antibody (anti-pCFTR, (Plog et al., 2010)) (D). The incubation of gCLCA2#2 transfected cells with the pre-absorbed gC2 antibody using the specific peptide for immunization did not detect any gCLCA2 protein (E). In contrast, the pre-absorption of the gC2 antibody with an irrelevant peptide did not reduce the signal intensity (F). After incubation of FFPE sections from chicken skin with the gC2 antibody, a prominent green signal was identified throughout all layers of the epidermis (G). This signal was not detected when identical sections were incubated with an irrelevant (anti-pCFTR) primary antibody. Alexa fluor 568 (B–F) and 488 (G–H)-conjugated secondary antibodies with DAPI counterstain (blue, G–H). Bars indicate 20 µm. Exposure times were 1 s for A, 190 ms (ms) for B–H and 333 ms for G–H (green channel). [file peerj-10-14202-s003.jpg]

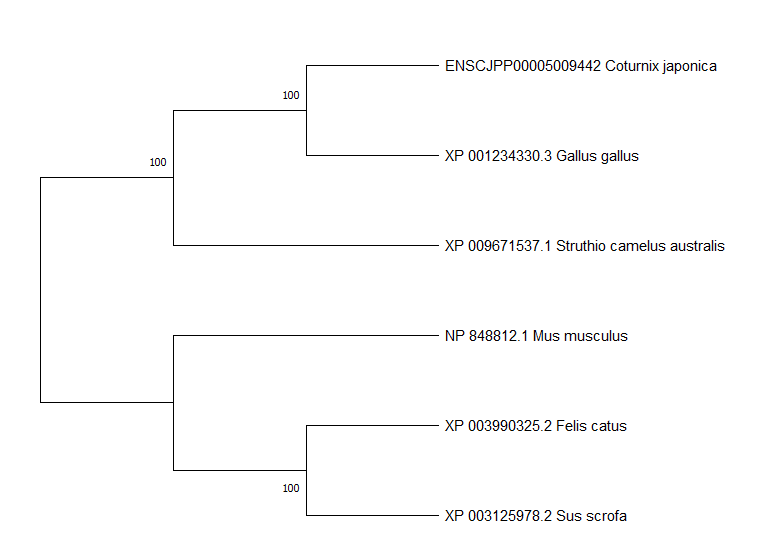

Supplement: Supplemental Information 6 — The evolutionary history was inferred by using the Maximum Likelihood method and JTT matrix-based model. The tree with the highest log likelihood (−6846.32) is shown. The percentage of trees in which the associated taxa clustered together is shown next to the branches. Initial tree(s) for the heuristic search were obtained automatically by applying Neighbor-Join and BioNJ algorithms to a matrix of pairwise distances estimated using the JTT model, and then selecting the topology with superior log likelihood value. A discrete Gamma distribution was used to model evolutionary rate differences among sites (5 categories (+G, parameter = 13.4417)). The rate variation model allowed for some sites to be evolutionarily invariable ([+I], 17.81% sites). This analysis involved 6 amino acid sequences. All positions containing gaps and missing data were eliminated (complete deletion option). Predicted signal peptide sequences were removed before analysis. There were a total of 873 positions in the final dataset. Evolutionary analyses were conducted in MEGA X. [file peerj-10-14202-s006.png]

## Original, uncropped Blots

Figure 3

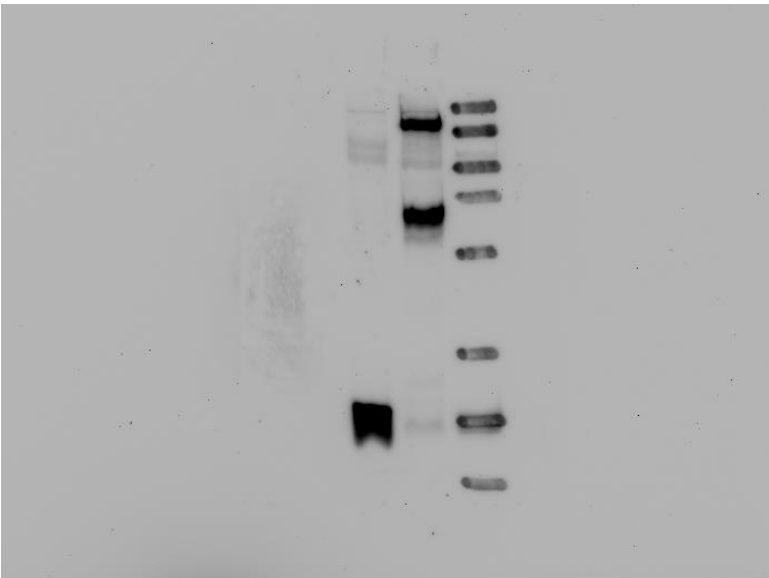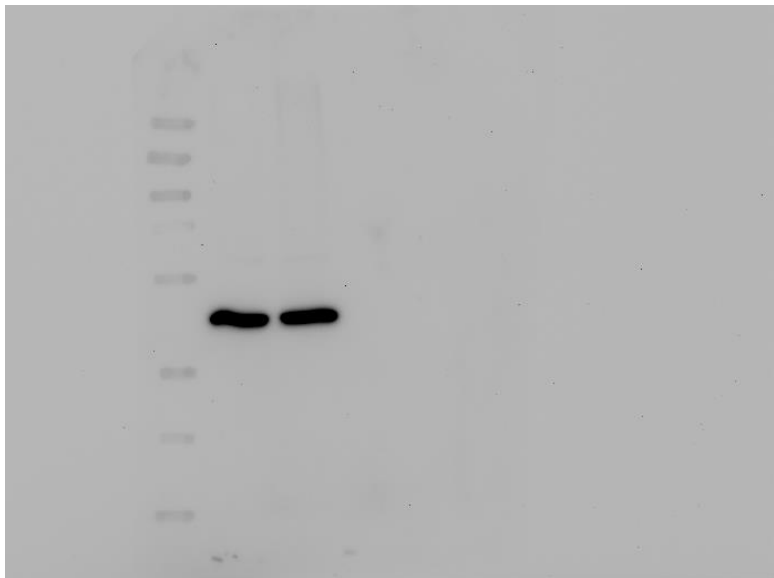

Figure 4

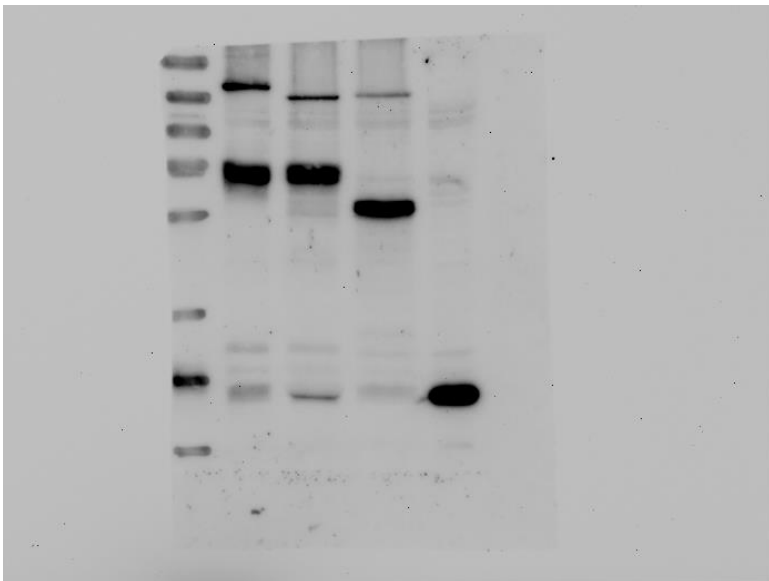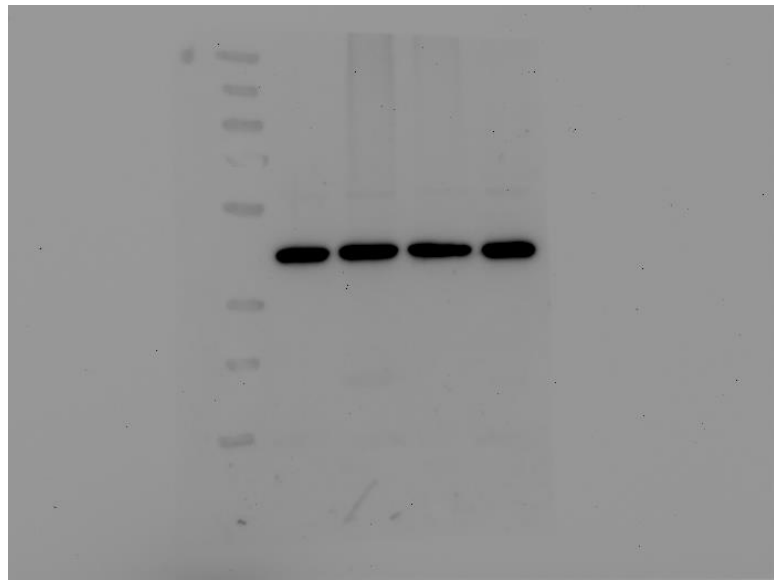

Supplement: Supplemental Information 7 [file peerj-10-14202-s007.pdf]
