# Supplementary material for: Genomic, biochemical and expressional properties reveal strong conservation of the CLCA2 gene in birds and mammals
Source: PeerJ. 2022 Nov 8;10:e14202. doi: 10.7717/peerj.14202 (PMC9651043; doi:10.7717/peerj.14202)
Supplement: Supplemental Information 4 — The comparison of the galline epitope, against the gC2 antibody was raised, with avian and porcine CLCA2 orthologues showed an expectation (e) value lower than the generally accepted threshold of 10−5 for possible cross-binding (McClain et al. 2017). Although the e values from feline and murine sequences were slightly above the threshold, convincing signals were detected in our study corresponding to the previous findings (Erickson, Gruber & Mundhenk, 2020), Braun et al. 2009, (Hamalainen et al., 2021). +: two sequences are similar but not highly similar, -: aa gap. McClain S. 2017. Bioinformatic screening and detection of allergen cross-reactive IgE-binding epitopes. Molecular nutrition & food research 61:1600676. Erickson NA, Nyström EE, Mundhenk L, Arike L, Glauben R, Heimesaat MM, Fischer A, Bereswill S, Birchenough GM, and Gruber AD. 2015. The goblet cell protein Clca1 (alias mClca3 or Gob-5) is not required for intestinal mucus synthesis, structure and barrier function in naive or DSS-challenged mice. PLOS ONE 10:e0131991. Braun J, Bothe MK, Mundhenk L, Beck CL, and Gruber AD. 2010. Murine mCLCA5 is expressed in granular layer keratinocytes of stratified epithelia. Histochem Cell Biol 133:285-299. 10.1007/s00418-009-0667-0 Hamalainen L, Bart G, Takabe P, Rauhala L, Deen A, Pasonen-Seppanen S, Karkkainen E, Karna R, Kumlin T, Tammi MI, and Tammi RH. 2021. The calcium-activated chloride channel-associated protein rCLCA2 is expressed throughout rat epidermis, facilitates apoptosis and is downmodulated by UVB. Histochem Cell Biol. 10.1007/s00418-021-01962-5. [file peerj-10-14202-s004.docx]

|  | **Epitope alignment** | **Expectation Value** | **Identities** | **Positives** |
| --- | --- | --- | --- | --- |
| **Quail**  epitope sequence  consensus sequence  quail sequence | WTAPGDDFDKGQAA  WTAPGDDFDKGQAA  WTAPGDDFDKGQAA | 7e^-09^ | 14/14(100%) | 14/14(100%) |
| **Ostrich**  epitope sequence  consensus sequence  ostrich sequence | WTAPGDDFDKGQAA  WTAPGDDFDKG_AA  WTAPGDDFDKGRAA | 2e^-07^ | 13/14(93%) | 13/14(92%) |
| **Turkey**  epitope sequence  consensus sequence  turkey sequence | WTAPGDDFDKGQA  WTAPGDDFDKGQA  WTAPGDDFDKGQA | 3e^-08^ | 13/13(100%) | 13/13(100%) |
| **Pig**  epitope sequence  consensus sequence  porcine sequence | WTAPGDDFDKGQA  WTAPG+DFD_GQA  WTAPGEDFDQGQA | 2e^-05^ | 11/13(85%) | 12/13(92%) |
| **Cat**  epitope sequence  consensus sequence  feline sequence | WTAPGDDFDKGQA  WTAPG+D+D_GQA  WTAPGEDYDQGQA | 2e^-04^ | 10/13(77%) | 12/13(92%) |
| **Mouse**  epitope sequence  consensus sequence  murine sequence | WTAPGDDFDKGQAA  WTAPG+DFD GQ  WTAPGEDFDQGQ | 6e^-04^ | 10/12(83%) | 11/12(91%) |
