# Supplementary material for: Genomic, biochemical and expressional properties reveal strong conservation of the CLCA2 gene in birds and mammals
Source: PeerJ. 2022 Nov 8;10:e14202. doi: 10.7717/peerj.14202 (PMC9651043; doi:10.7717/peerj.14202)
Supplement: Supplemental Information 5 [file peerj-10-14202-s005.docx]

In comparison to the reference genome assembly of the red junglefowl in the NCBI Genbank (GCA_000002315.5), the sequencing of three different *gCLCA2* clones concordantly identified a 2,792 base pair ORF with three nonsynonymous single nucleotide polymorphisms (nsSNPs): c.685T>A leading to p.229S>T, c.1342G>C leading to p.448D>H and c.2453T>C leading to p.818I>T. Additionally, 13 synonymous single nucleotide polymorphisms (sSNPs) were found: c.24G>C, c.30A>G, c.183G>A, c.189C>A, c.210A>G, c.615A>T, c.990G>A, c.1207T>C, c.1257T>A, c.1359G>A, c.1578C>T, c.1626C>T, c.2517A>G.
